# BTLA Expression in CLL: Epigenetic Regulation and Impact on CLL B Cell Proliferation and Ability to IL-4 Production

**DOI:** 10.3390/cells10113009

**Published:** 2021-11-04

**Authors:** Lidia Karabon, Anna Andrzejczak, Lidia Ciszak, Anna Tomkiewicz, Aleksandra Szteblich, Agnieszka Bojarska-Junak, Jacek Roliński, Dariusz Wołowiec, Tomasz Wróbel, Agata Kosmaczewska

**Affiliations:** 1 Laboratory of Genetics and Epigenetics of Human Diseases, Department of Experimental Therapy, Hirszfeld Institute of Immunology and Experimental Therapy, Polish Academy of Sciences, Weigl 12 Str., 53-114 Wroclaw, Poland; anna.andrzejczak@hirszfeld.pl (A.A.); anna.tomkiewicz@hirszfeld.pl (A.T.); 2 Department and Clinic of Urology and Oncologic Urology, Wroclaw Medical University, Borowska Str. 213, 50-556 Wroclaw, Poland; 3 Laboratory of Immunopathology, Department of Experimental Therapy, Hirszfeld Institute of Immunology and Experimental Therapy, Polish Academy of Sciences, Weigl 12 Str., 53-114 Wroclaw, Poland; lidia.ciszak@hirszfeld.pl (L.C.); aleksandra.szteblich@hirszfeld.pl (A.S.); agata.kosmaczewska@hirszfeld.pl (A.K.); 4 Department of Clinical Immunology, Medical University of Lublin, ul. Chodźki 4a, 20-093 Lublin, Poland; agnieszkabojarskajunak@umlub.pl (A.B.-J.); jacek.rolinski@gmail.com (J.R.); 5 Department and Clinic of Hematology, Blood Neoplasms, and Bone Marrow Transplantation, Medical University, Wybrzeże Ludwika Pasteura 4, 50-367 Wroclaw, Poland; dariusz.wolowiec@umw.edu.pl (D.W.); tomasz.wrobel@umw.edu.pl (T.W.)

**Keywords:** CLL, BTLA, IL-4, proliferation, epigenetic regulation, miR-155-5p

## Abstract

In our previous study, while chronic lymphocytic leukemia (CLL) cases showed higher levels of B and T lymphocyte attenuator (BTLA) mRNA compared to controls, lower BTLA protein expression was observed in cases compared to controls. Hence we hypothesize that micro RNA (miR) 155-5p regulates BTLA expression in CLL. In line with earlier data, expression of BTLA mRNA and miR-155-5p is elevated in CLL (*p* = 0.034 and *p* = 0.0006, respectively) as well as in MEC-1 cell line (*p* = 0.009 and 0.016, respectively). Inhibition of miR-155-5p partially restored BTLA protein expression in CLL patients (*p* = 0.01) and in MEC-1 cell lines (*p* = 0.058). Additionally, we aimed to evaluate the significance of BTLA deficiency in CLL cells on proliferation and IL-4 production of B cells. We found that secretion of IL-4 is not dependent on BTLA expression, since fractions of BTLA positive and BTLA negative B cells expressing intracellular IL-4 were similar in CLL patients and controls. We demonstrated that in controls the fraction of proliferating cells is lower in BTLA positive than in BTLA negative B cells (*p* = 0.059), which was not observed in CLL. However, the frequency of BTLA positive Ki67+ B cells in CLL was higher compared to corresponding cells from controls (*p* = 0.055) while there were no differences between the examined groups regarding frequency of BTLA negative Ki67+ B cells. Our studies suggest that miR-155-5p is involved in BTLA deficiency, affecting proliferation of CLL B cells, which may be one of the mechanisms responsible for CLL pathogenesis.

## 1. Introduction

Chronic lymphocytic leukemia (CLL) is the most common adult leukemia in Western countries, accounting for approximately 70% of all lymphoid leukemias. According to the latest data in the United States, the age-adjusted incidence is 4.1 per 100,000 inhabitants. Incidence increases with age to 12.8 per 100,000 in those who are 65 years and older. The mean age of CLL diagnosis is 72 years, and is 1.5–2 times more common in men than women [1]. CLL is characterized by the gradual accumulation of mature B cells expressing B-lineage-specific markers (CD19, CD20, CD23, and CD5 antigen) in lymphoid tissues, bone marrow, and peripheral blood (PB). The clonal B cells generated in CLL might be acquired at the hematopoietic stem cell stage. The leukemic transformation is initiated by specific genomic alterations increasing the resistance of B cells against apoptosis. A number of genetic and epigenetic abnormalities are observed in CLL patients such as deletions of chromosomes 13, 11, 17, and trisomy 12; numerous somatic and gene copy number mutations, mainly *NOTCH1, POT1, PTPN11, TP53, ATM* and also numerous epigenetic abnormalities related to micro-RNA regulation (reviewed in [1]).

Although CLL is a clinically and molecularly heterogeneous disease, both innate and adaptive dysfunction of the immune system increases the incidence of secondary malignancies and infections observed in patients. Defective neutrophil and NK cell function and decreased complement activity are characteristic of the disturbances in the innate immune system, whereas changes in adaptive immune response include cellular immunodeficiencies with hypogammaglobulinemia, decreased T cell function, and defects in antibody-dependent cellular cytotoxicity. Additionally, CLL cells modulate the phenotype and function of the immune system through a range of surface molecules and soluble factors [2]. Various studies indicate increased expression of the cytotoxic T lymphocyte antigen 4 (CTLA-4) molecule in the T and B cell compartment in CLL patients [3,4,5,6,7,8,9]. Our previous paper showed abnormal expression of another immune checkpoint molecule, B and T lymphocyte attenuator (BTLA) in CLL patients.

BTLA is a member of the immunoglobulin superfamily providing inhibitory signaling via the T cell receptor (TCR) or the B cell receptor (BCR), which in contrast to programmed cell death 1 (PD-1) and CTLA-4, binds to the member of TNF receptor superfamily namely to the herpes virus entry mediator (HVEM) [10,11,12]. As demonstrated in in vitro studies, BTLA has a direct negative activity on T cell proliferation and cytokine production. Moreover, BTLA is an inhibitory co-receptor of the BCR signaling pathway that, upon ligation with HVEM, attenuates B cell activation by targeting the downstream signaling molecules Syk and B cell linker protein [13]. BTLA inhibits CpG-mediated B cell functions (proliferation, cytokine production, and upregulation of co-stimulatory molecules) [14]. BTLA plays an important role in the maintenance of T cell tolerance, as disturbances of the BTLA-HVEM pathway have been shown to be involved in the pathogenesis of neoplastic disorders [15], infections [16] and autoimmune diseases [17]. Our recent study showed that in contrast to other types of cancer, BTLA protein level is significantly decreased in CLL B cells compared to normal lymphocytes, despite high expression of BTLA mRNA in CLL [18]. This observation points towards altered post-transcriptional regulation of BTLA in CLL cells. Therefore, we postulate that BTLA expression is regulated by microRNAs.

MicroRNAs (miRs) are a group of 19–23 nt long endogenously encoded RNAs which regulate gene expression at post-transcriptional level by complementary base-pairing with the target mRNA causing blockade of translation [19]. A single miR can regulate multiple target genes, and a single mRNA can be a target for multiple miRs. Over 30% to 90% of human genes are regulated by miRs [20]. Abnormal miR expression pattern has been linked to a broad range of human diseases including autoimmune diseases and various types of cancers. miRs can be involved in either carcinogenesis (oncomiRNA) and tumor suppression (anti-oncomiRNA) [21]. Different types of cancers can be characterized by their unique miR expression profile; this is especially true for CLL, since CLL cells can be easily distinguished from normal B cells based on miR profiling, making miR profiling a potential tool in the diagnosis of CLL [22,23]. The most extensively described miR in the context of CLL is the miR-15a/16-1cluster in the 13q14.3 region, which is commonly deleted in CLL (reviewed in [24]). However, many other miRs like miR-29, miR-181, miR-34a/34b/34c and miR-155 are associated with CLL risk and prognosis. Furthermore, genetic variations and epigenetic regulation of miRs are also associated with CLL pathogenesis [24]. Recently miRs are also considered as a potential target for CLL treatment (reviewed in [24,25]).

On the basis of a literature review and bioinformatic analysis we anticipate that miR-155-5p, which is highly upregulated in CLL [26] might be the factor regulating BTLA protein expression. This miR has been shown to be associated with BCR signaling disturbances in B cell neoplasms [24]. Moreover, *BTLA* gene was indicated as an miR-155-5p target [27] and on the mouse model it was shown that miR-155-5p regulates BTLA expression during CD4+ T cells activation [28].

The aim of this study is to verify our hypothesis about negative regulation of BTLA expression in B cells through epigenetic modulation by miR-155-5p. In addition, our goal is to evaluate the significance of BTLA deficiency in CLL B cells on proliferation capacity measured by Ki67 expression and IL-4 production, since IL-4 plays an essential role in the activation of mature B cells as well as providing survival signals and inhibiting apoptosis of splenic B lymphocytes [29].

## 2. Materials and Methods

### 2.1. Patients

Altogether 20 CLL patients and 15 healthy subjects were enrolled in this study. Patients were diagnosed based on criteria from the International Workshop on Chronic Lymphocytic Leukemia [29]. Detailed characteristics of patients is presented in Table 1. All participants were informed about the aim of the project and gave informed consent. These studies were approved by the local ethics committee (Wroclaw Medical University—KB—21/2010).

### 2.2. Peripheral Blood Mononuclear Cells (PBMCs) Isolation

Venous blood from healthy subjects and CLL patients were collected in CPTs containing sodium heparin anticoagulant (Greiner Bio-One, Kremsmunster, Austria). PBMCs were isolated by density gradient centrifugation using Lymphoflot reagent (Catalog #824012, Bio-Rad, Dreieich, Germany). Isolated PBMCs were frozen and refrozen directly before further investigation.

### 2.3. The Study of the Epigenetic Regulation of BTLA Expression by miR-155-5p

#### 2.3.1. Cell Culture and Transfection

Isolated PBMCs were suspended in basic OPTI-MEM medium (Catalog #11058021, Gibco Paisley, UK) and seeded into a 24-well plate for 1 × 10^6^ cells per well and incubated for 24 h. Next day, cells were transfected with 10 pmol miR-155-5p inhibitor (IN) (Catalog #4464084, Invitrogen, Waltham, MA, USA) or negative control (NC) (Catalog #4464076, Invitrogen, Waltham, MA, USA) using Lipofectamine RNAiMAX Transfection Reagent (Catalog #13778030, Invitrogen, Van Allen Way, Carlsbad, CA, USA) according to the manufacturer’s protocol. Then plate was centrifugated for 30 min at 1000 rpm at 37 °C to place suspended cells on the bottom of well [30]. After 24 h of incubation cells were harvested and used for further investigations. Cells were cultured in humidified 5% CO_2_ incubator at 37 °C. Additionally, an equivalent experiment was performed on the MEC-1 cell line. The transfection procedure was the same as for CLL patients and controls cell.

#### 2.3.2. miR-155-5p Expression Determination

MicroRNA was isolated from PBMCs and MEC-1 cells using miRNA Mini Kit (Catalog #SY391210, Syngen Biotech, Wroclaw, Poland), according to the manufacturer’s instructions. The cDNA templates were prepared from miRs using TaqMan Advanced miRNA cDNA Synthesis Kit (Catalog #A28007, Applied Biosystems, Van Allen Way, Carlsbad, CA, USA). RT-qPCR reactions were performed using TaqMan Universal PCR Master Mix, no AmpErase UNG (Catalog #4324018, Applied Biosystems, Warrington, UK) with miR-155-5p TaqMan Advanced MicroRNA Assay (Catalog #A25576, 477927_mir, Applied Biosystems, Pleasanton, CA, USA). The miR-361-5p (Catalog #A25576, 478056_mir, Applied Biosystems, Pleasanton, CA, USA) and miR-186-5p (Catalog #A25576, 477940_mir, Applied Biosystems, Pleasanton, CA, USA) were used as housekeeping genes. The experiment was carried out in duplicate. Quantitative miR expression data were acquired using ViiA7 Real-Time PCR system (Applied Biosystems, Singapore). The relative miR-155-5p expression levels (relative expression units—RU) were determined using the comparative Ct (2^−ΔΔCt^) method, and median expression in HC was used as calibrator.

#### 2.3.3. Determination of BTLA mRNA Expression

Total RNA was isolated from PBMCs and MEC-1 cells during procedure of miR isolation using miRNA Mini Kit (Catalog #SY391210, Syngen Biotech, Wroclaw, Poland), according to the manufacturer’s instructions. The cDNA templates were prepared from total RNA using High-Capacity cDNA Reverse Transcription Kit (Catalog #4368814, Applied Biosystem, Vilnius, Lithuania). RT-qPCR reactions were performed using TaqMan Universal PCR Master Mix, no AmpErase UNG (Catalog #4324018, Applied Biosystems, Warrington, UK) with BTLA TaqMan Gene Expression Assay (Catalog #4331182, Hs00699198_m1, Applied Biosystems, Pleasanton, CA, USA). The ACTB (Catalog #4331182, Hs03023943_g1, Applied Biosystems, Pleasanton, CA, USA) and GAPDH (Catalog #4331182, Hs02786624_g1, Applied Biosystems, Pleasanton, CA, USA) were used as housekeeping genes. The experiment was carried out in duplicate. Quantitative mRNA expression data were acquired and analyzed using ViiA7 Real-Time PCR system The relative BTLA mRNA expression levels (RU) were determined using the comparative Ct (2^−ΔΔCt^) method and median expression in HC was used as calibrator.

#### 2.3.4. Assessment of BTLA Protein Expression

After the culture with miR-155-5p inhibitor (IN) or negative control (NC) (as described above), the cells were washed and aliquoted into tubes for further surface staining of CD19, and BTLA with MoAbs conjugated with fluorochromes according to standard protocols (detailed procedure below). Directly after immunostaining, the cells were washed and analyzed by flow cytometry using a FACScan cytometer equipped with Cell Quest software (BD Bioscience, San Diego, CA, USA). At least 50,000 events per sample were analyzed in each experiment. The percentages of BTLA+ cells and mean fluorescence intensity (MFI) value expressed in arbitrary units (AU) were determined in subset of CD19+ lymphocytes.

### 2.4. The Study on the Influence of BTLA Expression on Cell Function

#### Assessment of B Cell Capacity to IL-4 Secretion and Proliferation

All experiments were carried out on PBMCs by labeling using the following monoclonal antibodies: CD19-PerCP (Pharmingen, San Diego, CA, USA), BTLA-PE (Becton Dickinson, Biosciences, San Diego, CA, USA), IL-4-FITC (Pharmingen, San Diego, CA, USA) or Ki67-FITC (Pharmingen, San Diego, CA, USA), and the appropriate isotype controls.

For induction of intracellular expression of IL-4 cytokine and proliferation marker Ki67, the thawed PBMCs were incubated with polyclonal stimulators in short-term cultures. Briefly, the cells were suspended at 1 × 10^6^ PBMCs/mL in RPMI 1640 medium (Gibco, Paisley, UK) supplemented with 10% fetal calf serum (Flow Labs, UK), L-glutamine (Gibco Invitrogen, São Paulo, SP, Brasil), and 50 g/mL gentamycin and cultured with 25 ng/mL phorbol 12-myristate 23-acetate (PMA)(Sigma-Aldrich, Merck KGaA, Darmstadt, Germany) and 1 µg/mL ionomycin (Ion) (Sigma-Aldrich, Merck KGaA, Darmstadt, Germany) in the presence of 10 µg/mL brefeldin A (BFA, protein transport inhibitor)(Sigma-Aldrich, Merck KGaA, Darmstadt, Germany) for 4 h at 37 °C in a humidified atmosphere containing 5% CO_2_. The cultured cells were next stained with anti-CD19 and anti-BTLA monoclonal antibodies (MoAbs), and then fixed and permeabilized with the Fixation/Permeabilization Buffer Set (eBioscience, San Diego, CA, USA) according to the manufacturer’s instructions. The efficacy of permeabilization was determined by uptake of trypan blue. Following washing, the cells were incubated for 30 min at 4 °C with anti-IL-4 or anti-Ki67 MoAbs conjugated with fluorochromes. Isotype-matched control antibodies were used to confirm expression specificity.

Directly after immunostaining, the cells were washed and analyzed by flow cytometry using a FACScan cytometer equipped with Cell Quest software (BD Bioscience). We analyzed the proportions of IL-4+ or Ki67+ cells within both CD19+BTLA+ and CD19+BTLA^−^ cells. At least 50,000 events per sample were analyzed in each experiment. The gating strategy is presented in Figure S1 in Supplementary Materials.

### 2.5. Statistical Analysis

Statistical analyses of the clinical data and laboratory findings were conducted using Statistica 10.0 software (TIBCO Software Inc., Palo Alto, CA, USA). For clinical parameters of CLL patients, the mean values and standard deviation (SD) are presented. Median values, the interquartile ranges were calculated additionally for all other variables. All collected data were examined for normal distribution using the Shapiro-Wilk test. For normally distributed data, the comparisons between studied groups were performed using the Student *t*-test for independent samples. In case of a non-normal distribution, the Mann-Whitney *U* test for comparison between groups was used. To test the effects of stimulation as well as effects of miR155-5p inhibition, the Student *t*-test for dependent samples and the non-parametric Wilcoxon signed-rank test were applied. In all analyses, differences were considered significant when *p* ≤ 0.05.

## 3. Results

### 3.1. The Study of the Epigenetic Regulation of BTLA Expression by miR-155-5p

#### 3.1.1. In Silico Analysis of miR-155-5p and BTLA Interaction

**Figure 1:**
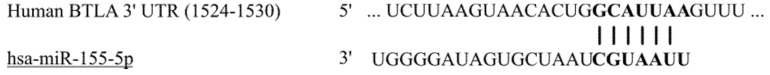
Predicted binding site of miR-155-5p in the 3′UTR of *BTLA* gene.

We selected miR-155-5p as a potential regulator of BTLA expression using the miR target gene prediction tool mirDIP (http://ophid.utoronto.ca/mirDIP/index.jsp#r, accessed on 30 November 2020). The 3′UTR region of the human BTLA mRNA was predicted to be the target of miR-155-5p. Our bioinformatic analysis of potential miR-155-5p binding sites within *BTLA* gene sequence revealed presence of 7mer-A1 canonical motif located within *BTLA* 3′UTR region (Figure 1). Canonical 7mer-A1 motif is created by an exact match to positions of 2–7 nucleotide of the mature miRNA followed by an A. The sequences of predicted site: 5′-GCAUUAA-3′.

#### 3.1.2. The Study of the Epigenetic Regulation of BTLA Expression by miR-155-5p in CLL Patients

##### mRNA BTLA Is Overexpressed in CLL Patients

**Figure 2:**
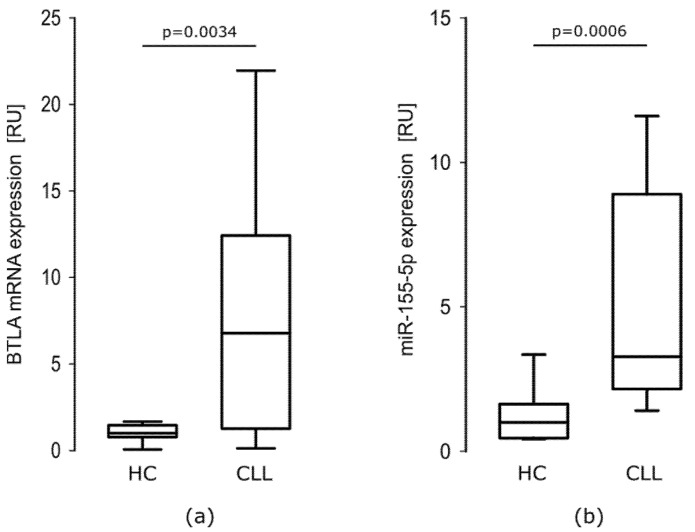
BTLA mRNA and miR-155-5p expression are elevated in CLL PBMCs. (**a**) BTLA mRNA is overexpressed in CLL PBMCs (*n* = 20) about 6-fold comparing to healthy controls (HC) PBMCs (*n* = 15) (*p* = 0.0034, Mann–Whitney test); (**b**) miR-155-5p expression level is upregulated about 3-fold in CLL PBMCs (*n* = 20) when compared to HC PBMCs (*n* = 15) (*p* = 0.0006, Mann–Whitney test). The central line shows the median, whiskers represent from the first quartile to the third quartile.

As we showed previously [18] mRNA BTLA expression was about 6-fold higher in CLL patients than in healthy controls (HC). In CLL cells, relative expression of BTLA mRNA ranged from 0.109–24.070 RU with a median of 6.776 RU. In HC cells, the levels of BTLA mRNA ranged from 0.052–1.671 RU with a median of 1000 RU. The difference between groups was statistically significant (*p* = 0.0034). As it is clearly seen in Figure 2a, the range of BTLA mRNA level is much wider in CLL patients than in HC.

##### miR-155-5p Is Overexpressed in CLL Patients

As previously shown in literature [26] the miR-155-5p is overexpressed in CLL patients compared to HC. In our study, in CLL PBMCs miR-155-5p relative expression ranged from 1.011–17.370 RU with a median of 3.274 RU, while in HC PBMCs, the levels of miR-155-5p ranged from 0.396–3.349 RU with a median of 1.000 RU. The difference between groups was statistically significant (*p* = 0.0006) (Figure 2b).

##### The Effect of miR-155-5p Inhibition on BTLA Protein Expression on B Cells

**Figure 3:**
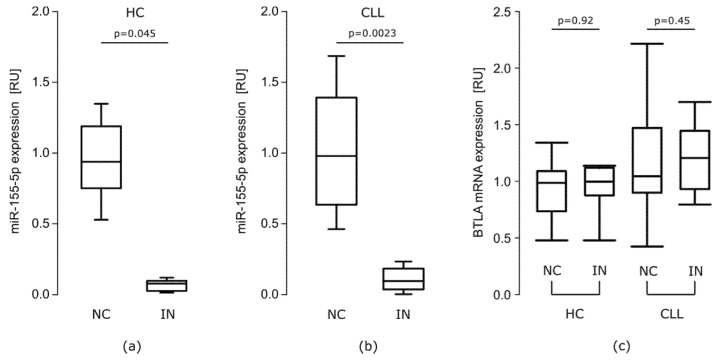
The effect of miR-155-5p inhibition. RT-qPCR analysis showed decreased expression level of miR-155-5p (**a**) in healthy controls (HC) PBMCs (*n* = 15) (*p* = 0.045, Mann–Whitney test) (**b**) in CLL PBMCs (*n* = 20) (*p* = 0.0023, Mann–Whitney test) after transfection with miR-155-5p IN. (**c**) Transfection with miR-155-5p inhibitor did not affect BTLA mRNA expression in both HC (*n* = 15) and CLL (*n* = 20). The central line shows the median, whiskers represent from the first quartile to the third quartile; IN- inhibitor, NC—negative control.

Transfection with miR-155-5p IN resulted in about 10-fold decrease in miR-155-5p level in both groups (IN vs. NC: HC median RU = 0.082, *p* = 0.045, CLL median RU = 0.088, *p* = 0.0023) (Figure 3a,b). However, miR 155-5p inhibition did not affect BTLA mRNA levels in either groups (Figure 3c).

**Figure 4:**
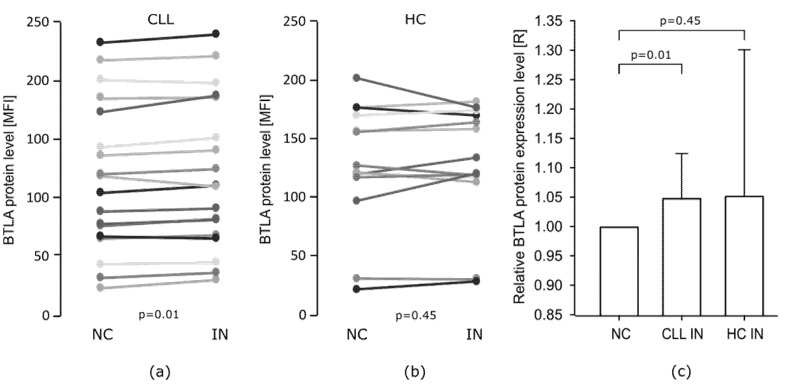
Effect of miR-155-5p inhibition on BTLA protein level in CD19+BTLA+ cells. (**a**,**b**) Flow cytometric analysis of BTLA protein level on CLL CD19+BTLA+ cells (*n* = 19) and healthy controls (HC) CD19+BTLA+ cells (*n* = 15) transfected with miR-155-5p NC or IN; (**c**) Inhibition of miR-155-5p increases BTLA protein level on CLL CD19+BTLA+. Relative protein expression level (R) was calculated for every patient as a fold change in MFI level after treatment with miR-155-5p IN compared to miR-155-5p NC (R = MFI of IN/MFI of NC). The graphs represent mean and SD of the results of all individuals in each group. IN—inhibitor, NC—negative control.

miR-155-5p inhibition increased BTLA protein expression level on CLL CD19+BTLA+ cells compared to cells transfected with NC. On average we detected a 5% increase in mean fluorescence intensity (MFI). This slight shift was observed in almost all CLL samples, which is statistically significant (*p* = 0.01) (Figure 4a,c). In the case of HC CD19+BTLA+ cells, we also noticed a similar slight increase in BTLA protein expression level, however, this difference was not statistically significant. Additionally, obtained results were more varied and we did not notice the same pattern of MFI increase as in the CLL group (Figure 4b,c).

#### 3.1.3. The Study of the Epigenetic Regulation of BTLA Expression by miR-155-5p in MEC-1 Cell Line

##### BTLA mRNA and miR-155-5p Is Upregulated in MEC-1 Cells

**Figure 5:**
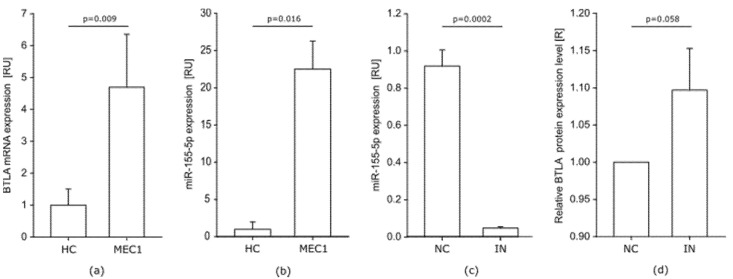
MEC-1 cell line. (**a**,**b**) BTLA mRNA and miR-155-5p are highly overexpressed in MEC-1 cells compared to median RU values of healthy controls (HC); (**c**) Downregulation of miR-155-5p in MEC-1 cells after transfection with miR-155-5p IN; (**d**) Relative BTLA protein expression level in MEC-1 cells after transfection with miR-155-5p IN; (R) Relative protein level was calculated for every experiment in MEC-1 cell line (*n* = 3) as a fold change in MFI level after treatment with miR-155-5p IN compared to miR-155-5p NC, (R = MFI of IN/MFI of NC); IN- inhibitor, NC—negative control. The graphs represents mean and SD of the results of three experiments.

Similar to cells from CLL patients, MEC-1 cells are characterized by elevated levels of BTLA mRNA and miR-155-5p. We observed that BTLA mRNA level in MEC-1 cells is lower than the median level in CLL patients but still significantly higher than in HC. Compared to controls, BTLA mRNA is higher over 4-fold in MEC-1 cells (Figure 5a). On the other hand, miR-155-5p is much more overexpressed in MEC-1 cells than in HC. The miR-155-5p is overexpressed more than 20-fold in MEC-1 cells (Figure 5b), while in CLL patients the highest determined level was about 17 RU compared to HC.

##### Influence of miR-155-5p Inhibition on the BTLA Protein in MEC-1 Cells

Similar to cells from CLL patients, transfection of MEC-1 cells with miR-155-5p IN decreased miR-155-5p level significantly (Figure 5c). As in CLL cells, transfection with miR-155-5p had no influence on BTLA mRNA expression in MEC-1 cells (data not shown). Inhibition of miR-155-5p in MEC-1 cells resulted in a 10% increase of relative BTLA protein level on MEC-1 CD19+BTLA+ cells compared to cells transfected with miR-155-5p NC (Figure 5d). The observed shift in BTLA protein level in MEC-1 cells was twice as much as in CLL patients, while due to differences between results for particular experiments the increase of BTLA expression level strongly tended to be statistically significant (*p* = 0.0579).

### 3.2. The Study on the Influence of Abnormal BTLA Expression on B Cell Function

Per our previous study demonstrating lower BTLA protein expression in CLL B cells as compared to controls [18], we aimed to verify whether BTLA impairment might affect B cell response to stimulation and effector function in CLL, thereby playing a role in disease pathogenesis. Therefore, we compared a capacity for proliferation and secretion of IL-4 in both CLL BTLA positive and BTLA negative B cells after PMA stimulation, comparing the obtained results with those seen in healthy controls. We found that in B cell subpopulation both CLL patients and healthy donors did not differ significantly when comparing the median proportions of BTLA positive and negative IL-4 secreting cells (Table 2, Figure S2 in Supplementary Materials). Likewise, both groups exhibited statistically comparable frequencies of corresponding B cells secreting IL-4.

**Figure 6:**
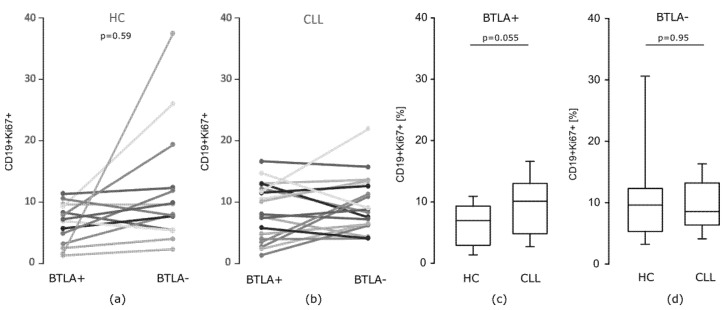
The proportion of BTLA positive and negative cells expressing Ki67 in B cell compartment. (**a**,**b**) The proportion of BTLA positive and negative cells expressing Ki67 in B cell compartment in particular individuals: (**a**) in healthy controls (HC) (*n* = 14), a lower Ki67 expression in BTLA positive B cells was observed (*p* = 0.059, test Wilcoxon), (**b**) while in CLL patients (*n* = 19), there were no statistically significant differences; (**c**,**d**) median proportion of Ki67-expressing cells in HC (*n* = 14) and CLL patients (*n* = 19) in BTLA positive (**c**), and BTLA negative B cells (**d**). The central line shows the median, whiskers represent from the first quartile to the third quartile.

Considering in vitro proliferative potential after PMA stimulation measured by Ki67 expression, as illustrated on Figure 6a, we found that in healthy individuals BTLA positive B cells expressed Ki67 in a lower proportion of cells compared to the BTLA negative B cell subset (*p* = 0.059). In contrast, in CLL there were no differences in frequency of BTLA positive and negative B cells exhibiting Ki67 (*p* = 0.277) (Figure 6b). Furthermore, we found that the median frequency of BTLA positive Ki67+ B cells in CLL was higher when compared to corresponding cells from healthy donors (*p* = 0.055) (Figure 6c); while, regarding BTLA negative Ki67+ B cell subset, we showed no statistically significant differences between the examined groups (Figure 6d). This part of our data demonstrates that, unlike IL-4 induction, a proliferative potential of CLL B cells might be dependent on BTLA protein expression level, thus indicating the involvement of BTLA molecule quantitative defect in promotion of B cell dysfunctional state.

## 4. Discussion

CLL is typically characterized by significant perturbations of the immune system, involving both innate and adaptive immune responses leading to immune suppression from an early stage. Dysfunction of the immune system in turn increases the incidence of secondary malignancies and infections, which represent the major cause of morbidity and mortality for CLL patients [2]. Growing evidence indicates that CLL cells modulate phenotype and functions of immune cells from the innate and adaptive immune system through a number of surface molecules and soluble factors. There has been a rising appreciation of the importance of co-stimulatory and co-inhibitory regulation pathways. Recently, we and others focused attention on the potential role of BTLA/HVEM pathway in CLL [15,18,31,32,33]. Our recent results showed an abnormal expression of BTLA in CLL patients [18]. We observed an elevated level of *BTLA* gene transcripts in peripheral blood B cells in CLL patients, which is in line with a recent study [33], despite showing, lower BTLA protein expression levels on PBMC B cells in CLL patients compared to PBMC B cells in controls [18]. Therefore, the aim of this study was to explore the mechanisms underlying an association of up-regulated *BTLA* gene induction with the decrease of BTLA protein expression observed by us in CLL [18] in terms of the epigenetic regulation of BTLA expression.

In mammalian genes the 3′UTR region contains highly conserved regions which are important for the regulation of transcriptional efficiency, polyadenylation and stability of the mRNA [34]. These functions are mediated by binding to mRNA interacting factors such as miRs [35]. Deregulation of miRs has been shown to affect the hallmarks of cancer, including sustaining proliferative signaling, evading growth suppressors, resisting cell death, activating invasion and metastasis and inducing angiogenesis [36]. It is especially true in CLL for which the earliest evidence of miR involvement in human cancer was provided by Dr Croce’s group from studies attempting to identify tumor suppressors at chromosome 13q14 region frequently deleted in CLL. The authors found that this region contains two miR genes, miR-15a and miR-16-1 [37], that both act as tumor suppressors to induce apoptosis by repressing Bcl-2, an anti-apoptotic protein overexpressed in malignant non-dividing B cells and many solid malignancies [38]. To date, several associations were described between certain miRs and cytogenic aberrations commonly found in CLL, as well as other prognostic factors [26].

MiR-155 is a well-known oncogenic miR that is overexpressed and associated with poor prognosis in many types of cancers [39], especially in numerous B-cell lymphomas [40,41] including CLL [37,42,43] suggesting that miR-155 contributes to lymphoma development [44]. This miR encoded within a region known as the B-cell integration cluster (*BIC, miR155HG*) is a critical regulator of posttranscriptional gene expression in B cells [45] Moreover, its overexpression is independently associated with poor prognosis. Relatively high-level expression of miR-155 in CLL has been also associated with expression of adverse prognostic markers, such as the ζ-chain associated protein of 70 kD (ZAP-70), unmutated immunoglobulin heavy chain variable region genes (IGHV), and/or deletions in 17p or 11q [26]. Evidence from animal models indicate that overexpression of miR-155 in transgenic mice induces polyclonal B-cell expansion, suggesting that miR-155 could enhance B-cell proliferation [46]_._ In line with this observation, it was shown that miR-155 in hematopoietic cells directly targeted Src homology-2 domain-containing inositol 5-phosphatase 1 (SHIP1). SHIP1 is a phosphatase that acts in opposition to kinases, that are integral to many signal transduction pathways. This inhibitory phosphatase suppresses surface immunoglobulin and BCR signaling. In mice, specific knockdown of SHIP1 in the hematopoietic system following retroviral delivery of a miR-155-formatted siRNA against SHIP1 resulted in a myeloproliferative disorder [47].

Transcriptome-wide miR-155 binding map studies revealed that BTLA mRNA is one of the targets of miR-155 in mouse T cells [27]. A subsequent study of another group on T cells brought evidence that miR-155 may be involved in the inhibition of BTLA expression. Liu et al. showed that BTLA mRNA might be a target of miR-155 during naïve CD4+ T cell activation in mice [28]. Both studies were performed on the T cell mouse model and to the best of our knowledge, there is no study confirming such interaction in a human model. Therefore, we decided to investigate BTLA/miR-155-5p interaction in human peripheral blood CD19+ cells of CLL patients. Our in silico analysis revealed that sequence of human *BTLA* gene contains MRE (miR recognition element) within its 3′UTR region. We predicted that in humans miR-155-5p interacts with *BTLA* sequence through 7mer-A1 canonical motif when in mice it has been shown that miR-155-5p binds to *BTLA* through 7mer-m8 canonical motif [28]. This difference may be due to the low degree of sequence homology of the *BTLA* gene between humans and mice. Therefore, confirmation of *BTLA*/miR-155-5p interaction in humans was needed.

Since, as mentioned above literature data showed overexpression of miR-155-5p in CLL and its important role in B cell lymphoproliferative disorders, we selected miR-155-5p as potential negative modulator of BTLA expression. In our study we confirmed previously published results (reviewed in [26]) that miR-155-5p is overexpressed in CLL patients as compared to controls. Moreover, we also confirmed our and others results [18,33] on another cohort of patients and controls that BTLA mRNA expression is significantly higher in CLL patients than in controls. With use of inhibitory siRNA targeting miR-155-5p we showed that decreasing of miR-155-5p level in CLL patients partially restored the BTLA surface protein level on B cells. Although the observed shift was slight, it was statistically significant and was present in almost all patients. A possible reason for this small observed changed is that lymphocytes are known to be hard to transfect. Although the transfection efficacy was low, we were able to observe significant changes in miR 155-5p expression and slight yet significant changes in the BTLA protein level on B cells. Another explanation might be that as many different miRs are upregulated in CLL cells, they can also be responsible for abnormal BTLA expression and in consequence could partially mask the effect of miR-155-5p inhibition. Until now only miR-32 has been described to regulate BTLA expression [48], thus, we cannot exclude the influence of other miRs on BTLA expression in CLL cells. Silencing of miR-155-5p did not have significant influence on BTLA expression in the case of HC B cells. This lack of significance might be the result of the much lower miR-155-5p and BTLA mRNA expression in healthy individuals, therefore miR-155-5p inhibition had no such effect on BTLA protein expression as seen in CLL cells.

Additionally, studies on MEC-1 cell line derived from B-chronic lymphocytic leu-kemia [49] confirmed the results obtained in CLL patients. Similar to CLL cells, MEC-1 cells are characterized by upregulated expression of BTLA mRNA and miR-155-p. 

In MEC-1 cells the effect of miR-155-5p inhibition on BTLA protein level was even stronger than median increase in CLL patients what can be the effect of slightly higher MEC-1 cells transfection efficacy. Altogether our results suggest that miR-155-5p is responsible for epigenetic regulation of BTLA expression in human B cells. However, additional studies are necessary to confirm BTLA-miR-155-5p interaction in humans. Furthermore, subsequent studies should also be performed on other subpopulations of immune cells.

In the current study, we also extended our preliminary data suggesting that decreased levels of BTLA protein expression in B cells of CLL patients might contribute to lowering the threshold for B cell activation and proliferation. To verify the above suggestion, we conducted a study to evaluate the ability of CLL B cells to proliferate and secrete IL-4 in response to polyclonal stimulation depending on BTLA expression. We found that secretion of IL-4, a growth and survival factor for CLL cells, appears to not be dependent on BTLA expression level, as fractions of IL-4 producing BTLA positive and BTLA negative B cells were found to be similar in all individuals studied. This is in line with previous findings that CLL B cells produce comparable amounts of IL-4 like healthy B cells and that the main source of IL-4 in CLL appears to be the T-cell population rather than B cells [50,51]. However, given the observation that CLL is a B-cell malignancy, the autocrine secretion of IL-4 might contribute to CLL pathogenesis as well.

Remarkably, our study also showed that proliferative rate of in vitro stimulated B cells expressing BTLA is increased and associated with impairment of BTLA protein level, thus strengthening the role of BTLA molecule as an attenuator of B cell activation and proliferation. After PMA stimulation, in CLL BTLA negative Ki67 expressing B cell subset was found to be expanded with the same level as BTLA positive proliferating (Ki67+) B cells subset, whereas in healthy controls the fraction of BTLA+Ki67+ B cell was lower. Furthermore, a proportion of BTLA+Ki67+ B cells in CLL patients was also seen higher confronting the corresponding healthy cells. One should note that our study was performed on the pooled CD19+ cells, including CLL cells as well, thus indicating that defect in BTLA protein expression resulting in dysfunctional state of B cells may be involved in pathogenesis of CLL. Although CLL B cells were historically considered to be resting and long-lived lymphocytes, increasing evidence indicates a much more active rate of cell birth and increase in proliferative capacity also in the periphery [52], an observation consistent with our present results.

## 5. Conclusions

Our studies suggest that miR-155-5p is involved in BTLA inhibition in CLL B cells, which may be one of the possible mechanisms responsible for CLL pathogenesis. Since we showed that inhibition of miR155-5p might partially restore BTLA expression in B cells BTLA/miR-155-5p axis can become a future target for personalized medicine in CLL patients.

## Figures and Tables

**Table 1:** Patients’ characteristics.

Clinical Parameter	Characteristics Value
Total number of Patients (n)	20
Gender (Female/Male)	6/14
Age	72.8 ± 9.46
Rai Stage	
0	9
1	6
2	2
3	1
4	2
WBC count (1 × 10^9^/L)	62.4 ± 50.9
Lymphocyte count (1 × 10^9^/L)	55.2 ± 48.7
Hb level (g/dL)	13.0 ± 1.5
Platelet count (1 × 10^9^/L)	185.7 ± 73.8
LDH (U/L)	168.5 ± 52.5
β2-microglobulin (mg/L)	3.8 ± 1.7

**Table 2:** Proportion of BTLA positive IL-4 expressing cells and BTLA negative IL-4 expressing cells in B cell compartment in CLL patients and healthy controls.

CD19+ Cells	Frequency of Positive Cells			
	CLL Patients		Healthy Controls	
	BTLA+IL-4+	BTLA-IL-4+	BTLA+IL-4+	BTLA-IL-4+
mean	56.2	47.2	48.4	52.3
median	54.5	41.5	56.4	51.2
SD	22.5	23.6	17.9	13.9
range	19.0–91.0	16.0–97.0	11.3–72.2	33.3–73.0
statistics	BTLA+IL-4+ vs. BTLA-IL-4+ *p* = NS		BTLA+IL-4+ vs. BTLA-IL-4+ *p* = NS	
	BTLA+IL-4+ CLL vs. HD, *p* = NS		BTLA-IL-4+ CLL vs. HD, *p* = NS	

NS—not significant.

## Data Availability

The data presented in this study are available on request from the corresponding author.

## Supplementary Materials

The following are available online at https://www.mdpi.com/article/10.3390/cells10113009/s1, Figure S1: Gating strategy for the evaluation of proportion of BTLA positive IL-4 or Ki67 expressing cells and BTLA negative IL-4 or Ki67 expressing cells in B cell compartment in CLL patients and healthy controls. Representative plots from a CLL patient demonstrating the analytic method for the identification of BTLA-positive and BTLA-negative B cells expressing IL-4 and ki67, Figure S2: The proportion of BTLA positive and negative cells expressing Ki67 in B cell compartment.
