# Supplementary material for: BTLA Expression in CLL: Epigenetic Regulation and Impact on CLL B Cell Proliferation and Ability to IL-4 Production
Source: Cells. 2021 Nov 4;10(11):3009. doi: 10.3390/cells10113009 (PMC8616199; doi:10.3390/cells10113009)
Supplement: Supplementary file 1 [file cells-10-03009-s001.zip › Figure S1. Gating strategy_functional assay.pdf]

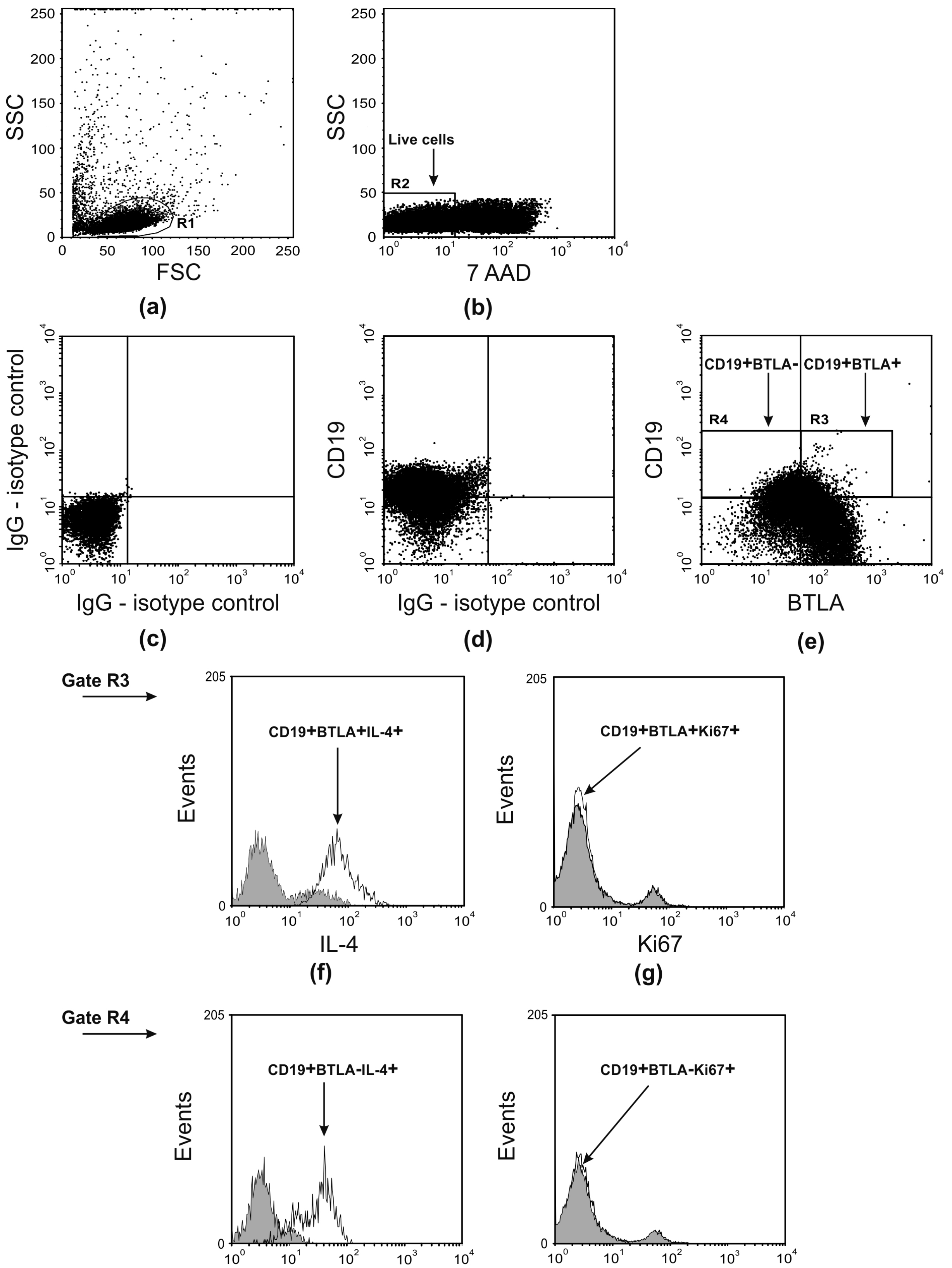

SSC
FSC
R1
(a)
SSC
7 AAD
Live cells
R2
(b)
IgG - isotype control
IgG - isotype control
(c)
CD19
IgG - isotype control
(d)
CD19
BTLA
CD19+BTLA-
CD19+BTLA+
R4
R3
(e)
Gate R3
Events
IL-4
CD19+BTLA+IL-4+
(f)
Events
Ki67
CD19+BTLA+Ki67+
(g)
Gate R4
Events
IL-4
CD19+BTLA-IL-4+
(i)
Events
Ki67
CD19+BTLA-Ki67+
(j)

Figure S1. Gating strategy for the evaluation of proportion of BTLA positive IL-4 or Ki67 expressing cells and BTLA negative IL-4 or Ki67 expressing cells in B cell compartment in CLL patients and healthy controls. Representative plots from a CLL patient demonstrating the analytic method for the identification of BTLA-positive and BTLA-negative B cells expressing IL-4 and ki67. (a) PBMCs were gated (R1) based on their FSC/SSC properties. (b) Gated PBMCs were then analyzed on SSC/7AAD profile for live and dead cells' discrimination; hence live cells (7-AAD-negative) were gated as region R2. (c-e) Final dot plots illustrate identification of BTLA-positive and BTLA-negative B cells. Gated live PBMCs were analyzed for CD19 and BTLA staining (dot plots: (c) cells stained with isotype control IgG for CD19; (d) cells stained with CD19 MoAb and isotype control IgG for BTLA; (e) double stained cells (for CD19 and BTLA); CD19+BTLA+ cells were gated as R3, while CD19+BTLA- cells were analyzed as R4). (f-i) Final histograms show IL-4 and ki67 fluorescence in the proportion of BTLA-positive (R3) and BTLA-negative (R4) B cells. Data were analyzed using Cell Quest software (BD Bioscience). FSC, forward scatter; SSC, side scatter; PBMC, peripheral blood mononuclear cell; MoAb, monoclonal antibody; BTLA, B and T lymphocyte attenuator; 7-AAD, (7-amino-actinomycin D) - Viability Staining Solution.
