# Supplementary material for: BTLA Expression in CLL: Epigenetic Regulation and Impact on CLL B Cell Proliferation and Ability to IL-4 Production
Source: Cells. 2021 Nov 4;10(11):3009. doi: 10.3390/cells10113009 (PMC8616199; doi:10.3390/cells10113009)
Supplement: Supplementary file 1 [file cells-10-03009-s001.zip › Figure S2.pdf]

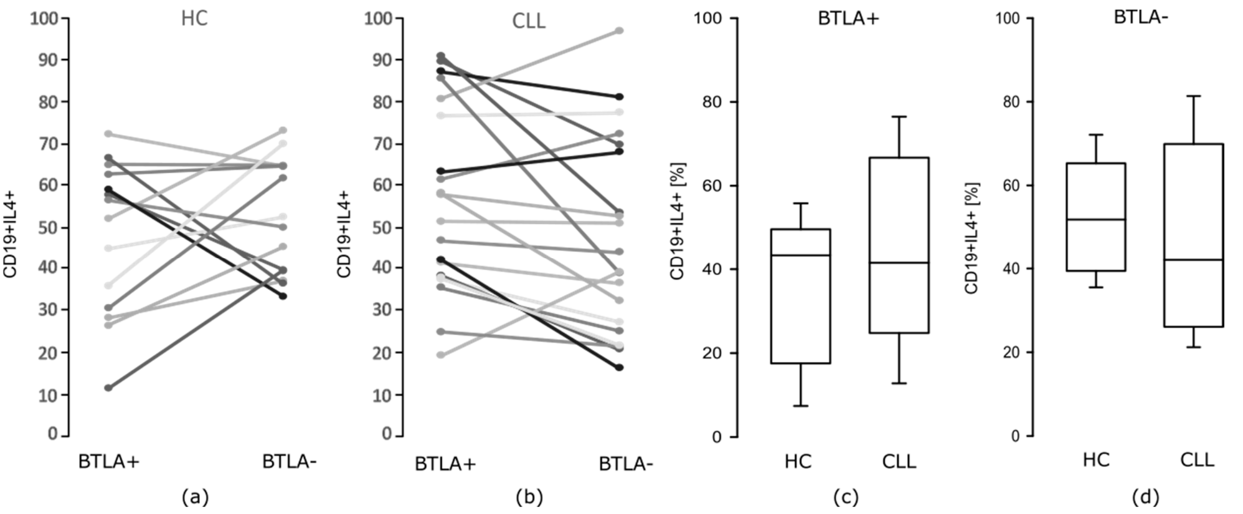


**Figure S2.** The proportion of BTLA positive and negative cells expressing Ki67 in B cell compartment.
